# Photonic Crystal Characterization of the Cuticles of *Chrysina chrysargyrea* and *Chrysina optima* Jewel Scarab Beetles

**DOI:** 10.3390/biomimetics3040030

**Published:** 2018-10-11

**Authors:** William E. Vargas, Esteban Avendano, Marcela Hernández-Jiménez, Daniel E. Azofeifa, Eduardo Libby, Ángel Solís, Cynthia Barboza-Aguilar

**Affiliations:** 1 Centro de Investigación en Ciencia e Ingeniería de Materiales, Escuela de Física, Universidad de Costa Rica, San José 2060-11501, Costa Rica; esteban.avendanosoto@ucr.ac.cr (E.A.); marcela.hernandezjimenez@ucr.ac.cr (M.H.-J.); daniel.azofeifa@ucr.ac.cr (D.E.A.); 2 Academia Nacional de Ciencias de Costa Rica, San José 1367-2050, Costa Rica; 3 Centro de Investigación en Ciencia e Ingeniería de Materiales, Escuela de Química, Universidad de Costa Rica, San José 2060-11501, Costa Rica; eduardo.libby@ucr.ac.cr; 4 Departamento de Historia Natural, Museo Nacional de Costa Rica, San José 749-1000, Costa Rica; asolis@museocostarica.go.cr; 5 Centro de Investigación en Estructuras Microscópicas, Universidad de Costa Rica, San José 2060-11501, Costa Rica; cynthia.barboza@ucr.ac.cr

**Keywords:** circular polarization, structural color, natural photonic material, photonic band gap, photonic crystal

## Abstract

A unified description involving structural morphology and composition, dispersion of optical constants, modeled and measured reflection spectra and photonic crystal characterization is devised. Light reflection spectra by the cuticles of scarab beetles (*Chrysina chrysargyrea* and *Chrysina optima*), measured in the wavelength range 300–1000 nm, show spectrally structured broad bands. Scanning electron microscopy analysis shows that the pitches of the twisted structures responsible for the left-handed circularly polarized reflected light change monotonically with depth through the cuticles, making it possible to obtain the explicit depth-dependence for each cuticle arrangement considered. This variation is a key aspect, and it will be introduced in the context of Berreman’s formalism, which allows us to evaluate reflection spectra whose main features coincide in those displayed in measurements. Through the dispersion relation obtained from the Helmholtz’s equation satisfied by the circular components of the propagating fields, the presence of a photonic band gap is established for each case considered. These band gaps depend on depth through the cuticle, and their spectral positions change with depth. This explains the presence of broad bands in the reflection spectra, and their spectral features correlate with details in the variation of the pitch with depth. The twisted structures consist of chitin nanofibrils whose optical anisotropy is not large enough so as to be approached from modeling the measured reflection spectra. The presence of a high birefringence substance embedded in the chitin matrix is required. In this sense, the presence of uric acid crystallites through the cuticle is strongly suggested by frustrated attenuated total reflection and Raman spectroscopy analysis. The complete optical modeling is performed incorporating the wavelength-dependent optical constants of chitin and uric acid.

## 1. Introduction

Reflection of light by cuticles of scarab beetles has attracted the attention of researchers since the beginning of the 20th Century, even more so after the circularly polarized nature of the reflected light was established [1]. Natural systems from bird feathers and butterfly wings to scarab cuticles, displaying what is referred to as structural color, were previously considered throughout the 19th Century by brilliant minds like the third and the fourth Lord Rayleigh [2,3] and Isaac Newton [4]. With the work of Robert Hook, optical microscopy contributes to a first approach to the structure of some of these iridescent natural systems [5]. Regarding scarab beetles, detailed descriptions of the self-assembled cuticular structures have been available since the use of electron microscopes [6]. Constructive interference between reflected light rays propagating through micron-sized structures composed of organized nano-sized elements is responsible for the color of these natural systems. Application of radiative transfer models has allowed characterization of the relationship between cuticular structures and corresponding reflection spectra. For instance, the iridescent green and purple colors of the elytra of the Japanese beetle *Chrysochroa fulgidissima* have been correlated with the presence of corresponding multilayered structures through its cuticle [7]. In this case, successive layers differ slightly in refractive index, and each one is assumed as optically homogeneous in the context of the radiative transfer model used to calculate reflection spectra consistent with the observed colors and linear polarization of the reflected light. Higher complexity optically anisotropic chitin-based natural arrangements have been considered in the last few decades, since the pioneering work of Neville and Caveney [8]. As in the cuticle of many scarab species, optical anisotropy is the result of a twisted or helical structure where each layer of chitin nanofibrils is slightly rotated with respect to the previous one until completion of several cycles throughout the cuticle; the thickness of these helicoidal stacks is usually between 10 and 20 μm. The circular polarization of the reflected light is determined by the chirality of the twisted structure. Most of the scarab beetles considered reflect left-handed circularly polarized light [9]. These kinds of structures, at larger scales, have also been found in crustaceans [10], in some fruits where the fibrils are made of cellulose [11], as well as in some plants’ leaves [12]. The role played by the circularly polarized reflected light in aspects such as thermal regulation, mating behavior, signaling, camouflage, etc., is a current theme of research [13,14], as well as the development of methods to produce functional polymer-based or cholesteric materials with similar twisted structures [15,16,17]. Regarding the twisted arrangements found through the cuticle of scarab beetles, structures characterized by a single pitch value with a small variation of the pitch from a certain depth through the cuticle and helicoidal arrangements with various pitches have been considered in connection with the spectral properties of the reflected light [18,19]. Both spectrophotometric- and ellipsometry-based analyses have been reported [20,21]. Several works have considered the optical properties of the cuticle of *Chrysina* scarabs. Photometric and ellipsometric measurements were reported by Goldstein for *Chrysina resplendens*, *Chrysina clypealis* and *Chrysina gloriosa* scarabs [22]. The silver-like appearance of *Chrysina chrysargyrea* has been qualitatively explained in terms of an assumed chirped structure through its cuticle [23], with no modeling or measurements. The cuticle surface of *C. gloriosa* has been studied by optical microscopy [24], and its polarizing properties analyzed through ellipsometric measurements [25,26]. Calculated reflection spectra by cuticles of *Chrysina aurigans* and *Chrysina optima* are reported [27], with no specification of the depth dependence of the pitch and with no comparisons with experimental measurements. Co- and cross-polarized measured reflection spectra by the cuticle of *Chrysina resplendens* have been compared with calculated ones with the incorporation of a scaled depth dependence of the pitch [28]. A photonic crystal characterization of the cuticle of *Hoplia coerulea* has been performed to correlate the presence of fluorophores with the density of states available for the photons emitted by fluorescence [29].

In the context of our research work, scanning electron images have shown that scarabs of the *Chrysina* genus are characterized by monotonously depth-dependent pitches, i.e., the spatial period gradually changes with the depth throughout the cuticle. For each specific species and varieties considered, when the depth dependence of the pitch is introduced in a radiative transfer model appropriate to describe the propagation of electromagnetic radiation through anisotropic uniaxial media, the calculated reflection spectrum is very similar to the measured one. This has been done for the silver-like and red *C. aurigans* and for the golden- and silver-like *C. chrysargyrea* beetles [30,31,32,33,34,35], including a photonic crystal (PC) characterization of *C. aurigans* varieties. These works introduced for the first time the explicit incorporation of a depth-dependent pitch in the radiative transfer formalism, dispersion in the optical constants of the involved materials, together with a PC characterization correlated with the presence of broad bands in the reflection spectra of these natural systems. 

In this work, we consider the optical properties of anisotropic twisted arrangements found in the cuticle of *Chrysina* scarab beetles through the application of Berreman’s radiative transfer formalism. Section 2 and Section 3 include details about the optical, morphological and composition characterization of the elytra whose optical properties and PC behavior will be considered, for three scarab beetles of the *Chrysina* genus: the golden-like *C. chrysargyrea*, the silver-like *C. chrysargyrea* and the silver-like *C. optima*. Spectrophotometric measurements of light reflection and scanning electron microscopy (SEM) were used to optically and morphologically characterize the cuticles of *Chrysina* scarab beetles. Additionally, frustrated attenuated total reflection of infrared light and Raman spectroscopy were used to carry out the compositional characterization of the elytra of *C. optima* specimens. Section 3 briefly describes the so-called 4 × 4 matrix approach, with some introductory applications focused on synthetic structures with a single pitch, a pitch linearly dependent on the depth through the cuticle and a pitch with a quasi-parabolic dependence on depth. The method followed to carry out a PC characterization of helicoidal arrangements is also described in this section, with application to a linearly graded arrangement and a structure with a quasi-parabolic dependence of the pitch on depth. The modeling of the photometric and photonic characterization of the chiral structures in nature includes graphics displaying both calculated reflection spectra and the depth dependence of the photonic band gap (PBG), its local limits and the effective spectral limits of the reflection band. Correlations between depth dependences of the band gaps and features of the reflection spectra are discussed.

## 2. Materials and Methods

### 2.1. Specimens and Sample Preparation

This research was developed with the approval of the Institutional Biodiversity Commission of the University of Costa Rica, resolution number 40. *C. optima* specimens used through this research were captured alive in the Tapantí National Park rainforest in Costa Rica. They were collected according to the permissions in the document “Resolución-SINAC-ACLAP-GMRN-INV-129-2016”. The collection was performed during the new moon period between April and May in 2017 and 2018. The specimens were attracted by means of a high intensity mercury lamp placed in from of a white screen faced towards the hillside of the mountain. After collection, the scarabs were put in a freezer to avoid degradation of the material. The *C. chrysargyrea* specimens were obtained through the material transfer agreement, from the collection of the former National Institute of Biodiversity (INBio), document number MTA-BP-001-11. Specimens were dried in a oven for preservation purposes. 

Samples for SEM were prepared following the method reported in [35] to determine the pitch of the elytra. For Raman spectroscopy and Fourier-transform infrared spectroscopy in frustrated attenuated total reflection configuration (FTIR-ATR), samples were prepared from the elytra upper surface of eight non-cured specimens carefully scraped with a steel blade to produce a fine micron-sized powder. Two reference samples were used: a low humidity and high purity uric acid (>99%) purchased from Fisher Scientific (Hampton, NH, USA) and a finely grained chitin powder extracted from shrimp shells. Raman spectra were recorded with an Alpha300R confocal Raman microscope (WiTec, Ulm, Germany) using a 785 nm excitation laser. Each spectrum was recorded with an integration time of 500 ms and an average of 100 scans. The infrared spectra were measured with a Universal ATR sampling accessory installed on a Frontier FIR/MIR spectrometer, both from Perkin Elmer (Waltham, MA, USA). Resolution was set to 0.5 cm^−1^ for an average of 50 scans per sample. The powder samples were covered with a gold foil and pressed against the diamond crystal, maintaining the same reading in the pressure gauge for each sample. 

### 2.2. Visual appearance

**Figure 1:**
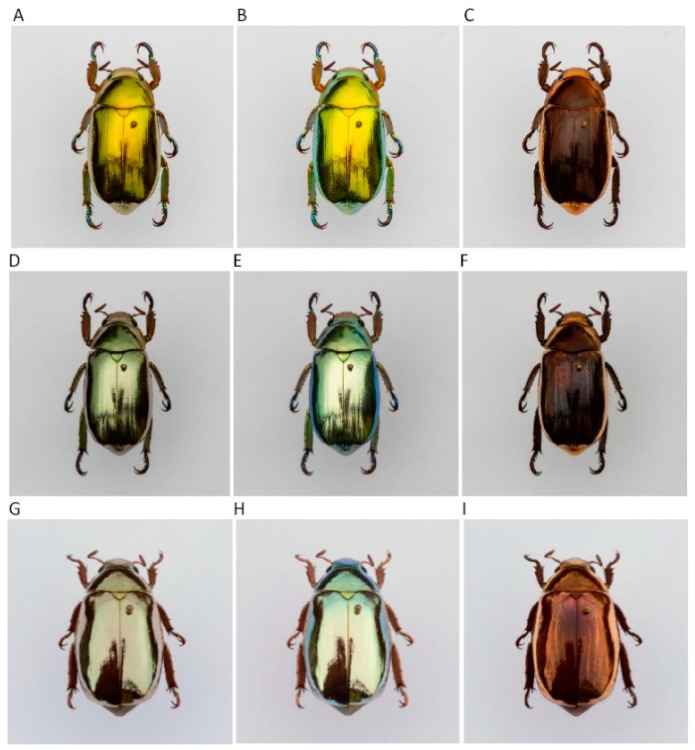
Appearance and circularly polarized light reflection from elytra of three jewel scarabs of the *Chrysina* genus. (**A**–**C**) Golden- and (**D**–**F**) silver-like *C. chrysargyrea*, and (**G**–**I**) silver-like *C. optima* specimens. The visual appearance of (**A**) golden *C. chrysargyrea*, (**D**) silvery *C. chrysargyrea*, and (**G**) silvery *C. optima* is shown on the left column. The middle column (**B**,**E**,**H**) shows photographs taken with a LHCP between the illuminated elytra and the objective of the camera. A RHCP was used for the photographs shown on the right column (**C**,**F**,**I**).

Figure 1 shows pictures of representative specimens of the jewel scarabs (golden- and silver-like *C. chrysargyrea* and *C. optima*) use in this study. Pictures where taken with a Nikon D7200 digital camera and AF-S 105 f/2.8 lens (Nikon Corporation, Tokyo, Japan). Exposure for all the images was normalized by spot-measuring the white-matte background to the side of the specimens and setting it to 1.33 f/stops above the meter reading. This exposure setting was chosen so none of the image RGB channels became oversaturated. The visual appearance of the scarabs is shown in Figure 1A,D,G. Figure 1B,E,H shows photographs taken when the light reflected by the elytra passes through a left-handed circular polarizer (LHCP) (HOYA PL-CIR, Hoya Corporation, Tokyo, Japan) before reaching the objective of the camera, and a right-handed circular polarizer (RHCP) (HOYA Pro1 MC PL-C, Hoya Corporation) was used to take the pictures shown in Figure 1C,F,I. As previously reported, the appearance of the scarabs when using a RHCP is determined by a background of non-coherent reflected radiation associated with the presence of defects (deviations from the gradual variation in the orientation of the chitin nanofibrils) through the chiral structure [31,33]. These defects are not related to the presence of a ripple structure superimposed on the broad band of reflection displayed for some of the *Chrysina* beetles like the golden *C. aurigans* and *C. chrysargyrea* [36]. When this non-coherent component of the reflected radiation is partially removed by the LHCP, the appearance of the cuticle is more intense. A visual comparison of the first two columns shows that most of the reflected radiation is left-handed circularly polarized. The reflection spectra were measured using an Avaspec 3648 fiber optic spectrometer (Avantes, Apeldoom, The Netherlands) in the wavelength range 300–1000 nm and using a halogen deuterium lamp AvaLight DHc (Avantes). A flat aluminum mirror was used as a reflectance standard STAN-SSH (Ocean Optics, Winter Park, FL, USA) for normalization of the measurements. An area of approximately 2 mm^2^ was illuminated with non-polarized normally incident radiation. Details on the procedure used to measure the spectra and to ensure reproducibility are reported elsewhere [30]. 

## 3. Results and Discussion

### 3.1. Measured Reflection Spectra

**Figure 2:**
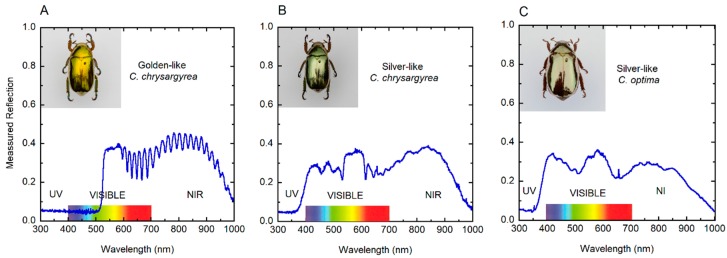
Measured reflection spectra of elytra from (**A**) golden-like *C. chrysargyrea*, (**B**) silver-like *C. chrysargyrea*, and (**C**) silver-like *C. optima* scarabs illuminated with normally incident non-polarized light. NIR: Near infrared; UV: Ultraviolet.

Figure 2 depicts the corresponding reflection spectra through the ultraviolet (UV), visible and near-infrared (NIR) wavelength ranges. The elytron of the golden *C. chrysargyrea* shows a spectrum with a reflection band from ≈525 to 1000 nm (containing a ripple structure beyond 600 nm), and it goes from 350 to 1000 nm for the silver-like scarabs whose spectra have a broad reflection band at NIR wavelengths. It also shows a flattened shoulder just after the reflection edge. For the silvery *C. chrysargyrea*, several peaks are depicted in the short wavelengths side of the visible range, with a plateau for wavelengths between 540 and 610 nm. The spectrum of the *C. optima* has additionally two broad peaks in the visible range. Similar measured spectra, in the wavelength range 450–1000 nm, have been reported for these two scarab beetles [37]. The presence of shoulders or plateaus is a consequence of a range of depths through the cuticle where the pitch is almost constant (see Section 3.5). Typically, the reflections spectra gradually decrease when approaching 1000 nm in wavelength.

### 3.2. Scanning Electron Microscopy Analysis and Morphology of the Cuticle

**Figure 3:**
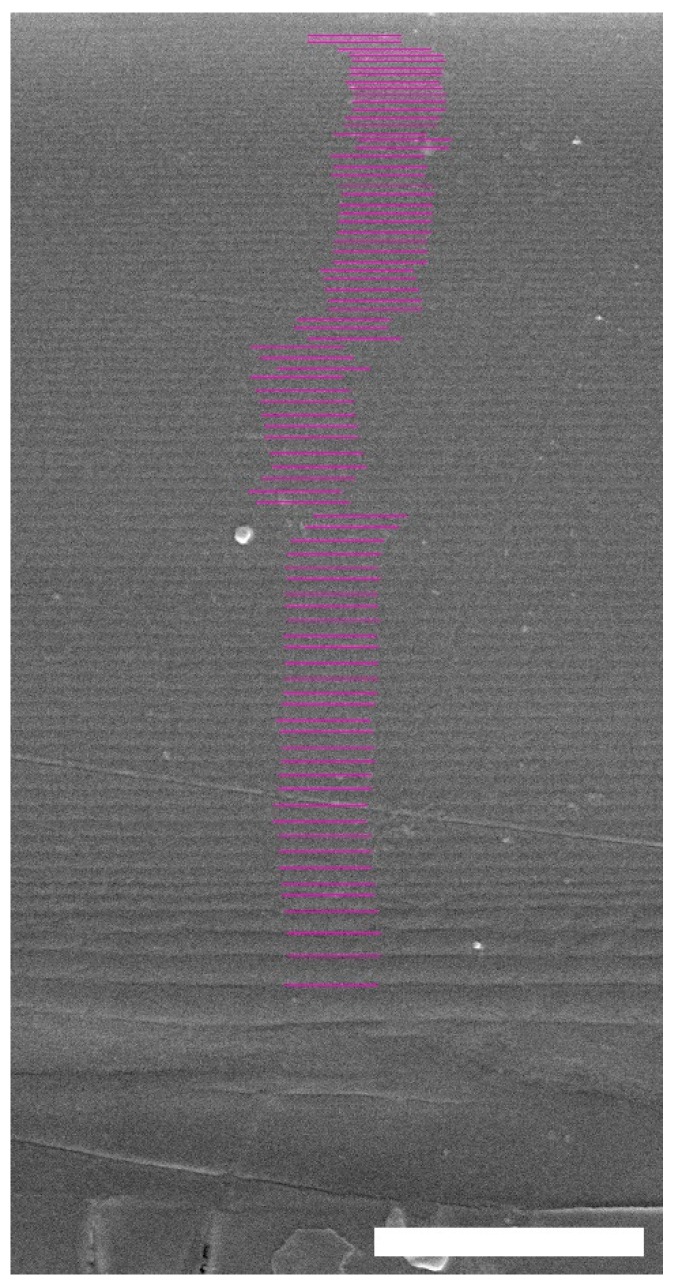
Scanning electron microscopy image of a cross-section through the cuticle of a *C. optima* scarab. The added violet lines, which cover a depth close to 17.2 μm, indicate those planes where the chitin fibrils are parallel to the cut of the twisted structure. Scale bar: 5 μm.

**Figure 4:**
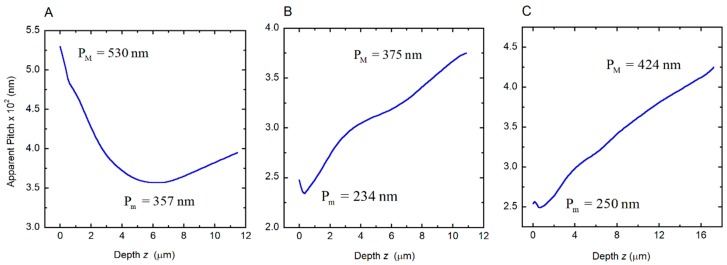
Depth dependences of the apparent pitches of the cuticles of (**A**) golden *C. chrysargyrea*, (**B**) silvery *C. chrysargyrea*, and (**C**) silvery *C. optima* scarab beetles. Maximum (*P_M_*) and minimum (*P_m_*) values of the apparent pitches are indicated.

Depth dependence of the apparent pitch was measured by SEM to characterize each twisted structure in the cuticles of the beetles under study. This was previously reported for the *C. chrysargyrea* scarabs [35]. Their apparent thicknesses are close to 11.5 and 10.9 μm for the golden and silvery *C. chrysargyrea* scarabs, respectively. The apparent thickness of the *C. optima*’s elytron is the largest one, ≈17.2 μm. Following a similar method as described in [35] to determine how the pitch of the *C. optima* changes with depth through the cuticle, Figure 3 shows a cut through the cuticle with the added violet lines coinciding with those planes where the chitin nanofibrils are parallel to the cut. The plane of the cut could be inclined with respect to the plane normal to the surface, with β as the angle of inclination. Changes in the orientation of the chitin nanofibrils, every 180°, can be correlated with the depth *z* through the cuticle, and as a consequence of this, the discrete depth dependence of the apparent pitch can be established. From this set of *z*-values, whose uncertainties are close to *δz* = 5 nm, a smoothed curve is obtained and used to interpolate when applying Berreman’s formalism. Determination of these parameters from SEM images allows us to depict the depth dependence of the apparent pitches (Figure 4) corresponding to the cuticles of the scarabs shown in Figure 1. The depth dependence of the golden-like *C. chrysargyrea* shows an extended quasi-parabolic behavior, and as commented on before, this fact is directly correlated with the ripple-structured broad band displayed in its reflection spectrum. The pitches of the silver-like beetles show small decreases near the surface of the structures, followed by monotonously increasing values showing some shoulders for certain ranges of depth. 

### 3.3. Composition

Chitin is a natural polymer with a low birefringence. Besides the twisted structure of chitin nanofibrils, the existence of a high birefringent substance embedded between the arrangement of chitin is required to approach the measured reflection spectra from modeling. The occurrence of crystallites of such a material through twisted structures of chitin nanofibrils in cuticles of *Chrysina* scarabs was established some time ago, particularly when considering *C. resplendens* and *C. optima* specimens. Our choice of uric acid for this strongly birefringent substance follows the original determinations of Caveney [38], who recognized the presence of absorption bands characterizing the UV spectrum of uric acid. Caveney’s slow extraction of uric acid, accompanied by loss of the metallic reflectivity, when using 70% alcohol or with 1% ammonia, is consistent with a polar and acidic substance (uric acid pKa1 = 5.4). There are many reports of the occurrence of uric acid in insect cuticles. For example, it is present in the white stripes of the armyworm *Pseudaletia separata* [39] and in the reflecting cuticle of the light organ of fireflies [40]. However, exists some controversy with regard to Caveney’s chemical extraction experiments performed in the 1970s that may not support this claim [7,41]. 

**Figure 5:**
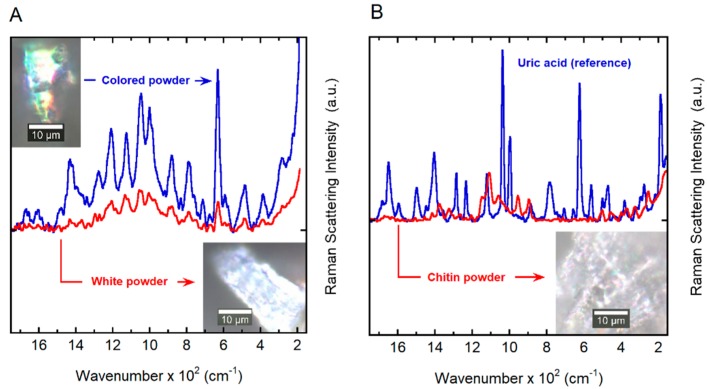
Spectral dependence of the Raman scattering intensity. (**A**) Raman spectra of white (lower-right inset) and colored (upper-left inset) powders obtained by scraping of the cuticles of *C. optima* specimens. (**B**) A reference spectrum of uric acid is shown together with the spectrum of chitin powder obtained from shrimp shells (inset). a.u.: Arbitrary units.

Initially, Raman and FTIR-ATR spectra were measured directly from the elytra for both non-cured samples obtained from recently captured specimens and cured samples from a collection. Cured samples showed a reduced Raman signal by a factor of 10 with respect to the fresh samples. In addition, the spectra showed a broadening and changes in position of the vibration modes. The FTIR-ATR spectra showed significant differences in water content (structural water embedded on the chitin arrangement) and degradation of organic compounds. To minimize those effects, the elytra upper surface of eight non-cured specimens was carefully scraped with a steel blade to produce a fine micron-sized powder like that shown in the insets of Figure 5A. The two reference samples were measured; chitin powder (red line) can be easily distinguished from that of the uric acid (blue line), as shown in Figure 5B. The chitin and uric acid powder standards used are in excellent agreement with previously reported studies [42,43]. Figure 5A shows two spectra measured from the powder of *C. optima* cuticle (PCOC); the sample obtained from eight elytra specimens presented two distinct particulates: (i) a white powder (lower-right inset in Figure 5A) that resembles chitin powder (inset in Figure 5B); and (ii) a colored particulate (upper-left inset in Figure 5A) that appears more crystalline than the other agglomerates judging from the narrow bands and strong signal measured in comparison with the white crystals. There is a distribution of these types of crystals obtained from the elytra cuticle as reported in [38]. Figure 5 shows the Raman bands for the PCOC sample at 497 (501)^c^, 630 (624)^ac^, 999 (897)^c^, 1002 (998)^ac^, 1043 (1060)^c^, 1045 (1037)^ac^, 1113 (1108)^c^, 1338 (1236)^c^, 1384 (1376)^c^, 1429 (1406)^ac^, 1607 (1596)^ac^ and 1648 (1650)^ac^ (in parenthesis, the position of the chitin (c) and uric acid (ac) standards; all data in relative units of cm^−1^). The band spectra are a close match for those of the uric acid standard; nonetheless, many of these vibration modes are also present in many other ring compounds [42]. 

Because the PCOC sample contains at least one known component, chitin, and an unknown number of other organic compounds, it was not possible to obtain an exact matching of the vibration modes with respect to the standards. Moreover, an exact reconstruction of the spectra from the standards is not possible due to the lack of information regarding the nature of the environment in which the molecules are embedded. The position of the vibration modes may vary depending on the chemical environment and composition of the samples. From the Raman analysis, we concluded that it is likely that PCOC may contain a uric acid-type compound and, thus, we will use the term “uric acid” to refer to it. 

**Figure 6:**
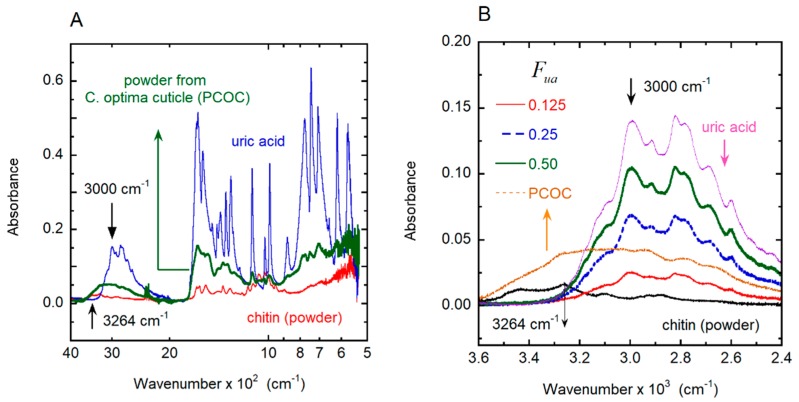
Spectral dependence of the infrared absorbance by chitin, uric acid, and powder from *C. optima* cuticle. (**A**) Infrared absorption of samples of chitin powder, uric acid and powder from the cuticle of a *C. optima* specimen (PCOP). (**B**) Infrared absorbance within a smaller wavenumber range, including the absorption of chitin samples containing different volume fractions of uric acid (*F_ua_*).

Additionally, absorbance measurements were performed by FTIR-ATR. Figure 6A shows the measured infrared absorbance spectrum of the two standards, uric acid and chitin; both coincide in their spectral details with those reported in the literature [44,45]. For wavenumbers between 500 and 1800 cm^−1^, both display absorption peaks with overlapping spectral positions. From 1800 to 2800 cm^−1^, the light absorption by chitin is negligible [46], with a decreasing absorption tail approaching 2800 cm^−1^. The full spectrum of the PCOC measured under the same conditions is also shown. At ≈3000 cm^−1^, uric acid presents a stretching vibration mode for the N–H bond [47] that it is not present in chitin. There are no vibration modes of chitin at this spectral position, and the perpendicular stretching vibration mode for the N–H bond is reported in the vicinity of 3264 cm^−1^ [44,47], as indicated in Figure 6A. Figure 6B shows the absorption spectra of the uric acid standard, chitin powder standard, the PCOC sample and three different homogeneous mixtures of uric acid and chitin (standards) for volume fractions of uric acid of 0.500, 0.250 and 0.125. In the range 2400–3600 cm^−1^, the absorbance contributions of uric acid and the natural polymer can be distinguished. At ≈3000 cm^−1^, the chitin standard does not present a vibration mode different from the pure uric acid, as shown in Figure 6B. The PCOC sample shows, in the vicinity of 3000 cm^−1^, a strong absorbance that resembles the convolution of chitin and uric acid. The additional measurements in the infrared strongly suggest the presence of a uric acid-type compound, in agreement with the Raman spectra. Without a careful chemical extraction, it is not possible to confirm that the compound is pure uric acid. However, from the vibration spectra presented, it is possible to conclude that the compound is a ring compound very closely associated with uric acid.

A key point of this discussion is the birefringence nature of the compound associated with the metallic-like structural color of these beetles [30,31,32,33,34,35], which should be deposited within the chitin structure. There are many reports of doping or functionalized uric acid [48,49,50,51,52], but not all of them present a birefringence [48]. The chemical extraction of the PCOC sample was attempted by using the method suggested by Caveney [38]. The method was shown to be an extremely slow reaction, and limitations were found regarding a sufficient amount of mass to perform a nuclear magnetic resonance (NMR) analysis. This will be a subject of future investigations, in the case that a sufficient amount of sample is produced for analysis. By using the absorbance values at 3000 cm^−1^ and by assuming that the uric acid-type compound has a mass density equal to the pure uric acid, we calculated the average volume fraction for the cuticle of a *C. optima* specimen by linear regression as *F_ua_* ± Δ*F_ua_* = 0.17 ± 0.01. We neglected any other source of error in this estimation, because of the difficulty of assessing this in terms of statistical methods; that is, the limitations in the preparation of more samples for comparison due to the lack of specimens. This value will be used in the context of Berreman’s formalism [53] to evaluate the average values of the ordinary and extraordinary refractive indices of the structure, as well as its effective birefringence. It is important to mention that the PCOC was taken from non-cured samples obtained from recently captured specimens, and the amount of water present inside the structure is evident because of the increase in the absorbance at the end of the spectral range (Figure 6B). Furthermore, the use of the calculated volume fraction (*F_ua_* = 0.17) on the calculation of the reflection spectrum allows us to obtain a good fitting with the measured one (see Figure 2C and Figure 13A). This fact can be used to assess the quality of the method followed to determine the volume fraction of uric acid. The only drawback of this method is the difficulty in obtaining sufficient “fresh” samples for other species. 

### 3.4. Modeling Synthetic Reflection Spectra with Berreman’s Formalism

Regarding the modeling of photometric measurements, Berreman’s formalism is suitable to describe the propagation of electromagnetic radiation through uniaxial anisotropic media [53]. In this approach, a plane parallel anisotropic structure, whose thickness is *H*, is divided into successive thin layers, each one of thickness *h*. The *h*-value is set small enough to ensure numerical convergence in the reflection spectra: *H* = *Nh*, with *N* the number of thin layers. The continuous tangential components of the electric and magnetic fields at successive interfaces of these thin layers are related through the layer transfer-matrix *P* = exp(−*ik_o_*Δ*h*), where *k_o_* = 2*π/λ* is the wavenumber of the incident radiation, *λ* its wavelength and with the elements of Berreman’s propagation Δ matrix depending on the angle of incidence (φ), the refractive index of the medium covering the structure (*n_c_*), the refractive index of the substrate beneath the chiral structure (*n_s_*), the chirality of the helical arrangement (*γ*), the extraordinary and ordinary refractive indices (*n_e_* and *n_o_*, respectively) or the respective dielectric functions (*ε_e_* = *n_e_*^2^ and *ε_o_* = *n_o_*^2^), as given elsewhere [35]. The birefringence is given by Δ*n* = *n_e_* − *n_o_* and the average refractive index is *n_av_* = (*n_e_* + *n_o_*)/2. The Δ matrix also depends on the angle *ϕ* specifying the orientation of the polarizable units: cholesteric macromolecules or chitin nanofibrils, for instance. This angle depends on the pitch *P* through the relation *ϕ* = 2*πz/P*, where *P* can be a constant (*P* = *P_o_*) or dependent on *z*, the depth through the twisted arrangement measured from its illuminated interface (*P* = *P*(*z*)). Our computational implementation of this approach follows the formulation of Wöhler et al. [54] to evaluate the layer transfer-matrices and that of Brink et al. [19] to compute the co- and cross-polarized complex amplitude reflection coefficients for circularly polarized light [55]: *r*_LL_ and *r*_RR_, and *r*_LR_ and *r*_RL_, respectively; and the corresponding reflections: *R*_LL_ = |*r*_LL_|^2^, *R*_RR_ = |*r*_RR_|, *R*_LR_ = |*r*_LR_|^2^ and *R*_RL_ = |*r*_RL_|^2^. Under non-polarized incident radiation, the total reflection *R*_T_ is calculated as follows: *R*_T_ = (*R*_LL_ + *R*_LR_)/2 + (*R*_RL_ + *R*_RR_)/2. This means that when doing the measurements and the calculations, there are no polarizers between the reflecting surface and the objective of the sensor receiving the reflected light. In the Wöhler approach, successive layer transfer-matrices are evaluated from the eigenvalues of Berreman’s propagation Δ matrix. 

We will apply Berreman’s formalism following a gradual procedure, in terms of how the pitch depends on depth through the cuticle, going from the simplest case to those found in nature. In this regard, our results can be compared with the literature on synthetic structures. On the other hand, this approach allows us to show the correlation of some of the optical, structural and PC properties with features of the reflection spectra.

**Figure 7:**
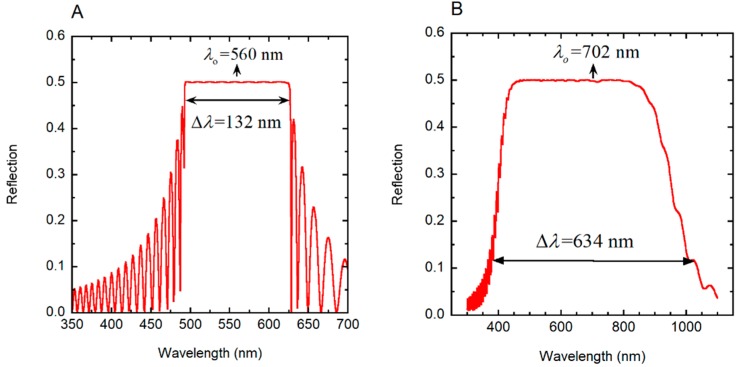
Reflection by chiral structures when normally illuminated with non-polarized light. (**A**) Total reflection spectrum of a cholesteric liquid crystal with the following specification: *P_o_* = 330 nm, *H* = 16*P_o_*, Δ*n* = 0.40 and *n_o_* = 1.5 [56]. (**B**) Reflection spectrum of a linearly graded left-handed twisted cholesteric liquid crystal, whose pitch is given by *P*(*z*) = *P_m_* (1 + *az/H*) with *a* = 0.458, *H* = 24*P_m_*, *P_m_* = 240 nm, Δ*n* = 0.40, and *n_o_* = 1.5. Normal illumination of non-polarized radiation is assumed.

First, we consider the evaluation of the reflection spectrum of a single-pitch cholesteric right-handed liquid crystal (*γ* = 1) with the following specification: *P_o_* = 330 nm, *H* = 16*P_o_*, Δ*n* = 0.40 and *n_o_* = 1.5 (*n_e_* = *n_o_* + Δ*n*). The sample is normally illuminated (*φ* = 0) with non-polarized radiation, and the calculated total reflection spectrum is shown in Figure 7A. This system, with a high birefringence, has been considered by Hong et al. [56] with their calculations performed by Berreman’s formalism, as well as a finite element method. A very good agreement of our results with those reported (see Figure 4 in [56]) is provided, with coincidence in the number of peaks and their amplitudes at both sides of the reflection band. The optical band gap is centered at *λ_o_* = *n_av_P_o_* = 560 nm, with *n_av_* = 1.70 as the average refractive index and bandwidth of Δ*λ* = Δ*nP_o_* = 132 nm.

For wavelengths within the optical band gap, 50% of the incident radiation is reflected as right-handed circularly polarized light. The larger the birefringence, the higher the top of the reflection band, which approaches 50%, and the wider its width (see Figure 3 in [57]). Our second example corresponds to a linearly graded left-handed pitch (*γ* = −1) given by *P*(*z*) = *P_m_* (1 + *az*/*H*), with *P_m_* as the minimum value of the pitch, *a* = (*P_M_* − *P_m_*)/*P_m_* = Δ*P*/*P_m_*, where *P_M_* is the maximum value of the pitch (chirped structure). The following values are assumed: *P_m_* = 240 nm, *P_M_* = 350 nm, *a* = 0.458, *H* = 24*P_m_*, Δ*n* = 0.4 and *n_o_* = 1.60. By assuming normally non-polarized incident radiation, Figure 7B shows the calculated broad band total reflection spectrum. This is in agreement with the spectrum shown in Figure 6 in [56], which was calculated for a right-handed helical arrangement. The graded pitch widens the reflection band. Both reflection spectra, the reported and the calculated, must coincide for non-polarized incident light, although their circular polarizations correspond to those of the chiral structures. The values of *λ_o_* and Δ*λ* indicated in the figure were calculated as explained below, in the context of the PC characterization of the helicoidal stack. 

### 3.5. Photonic Crystal Characterization of Twisted Structures with Graded Pitches

**Figure 8:**
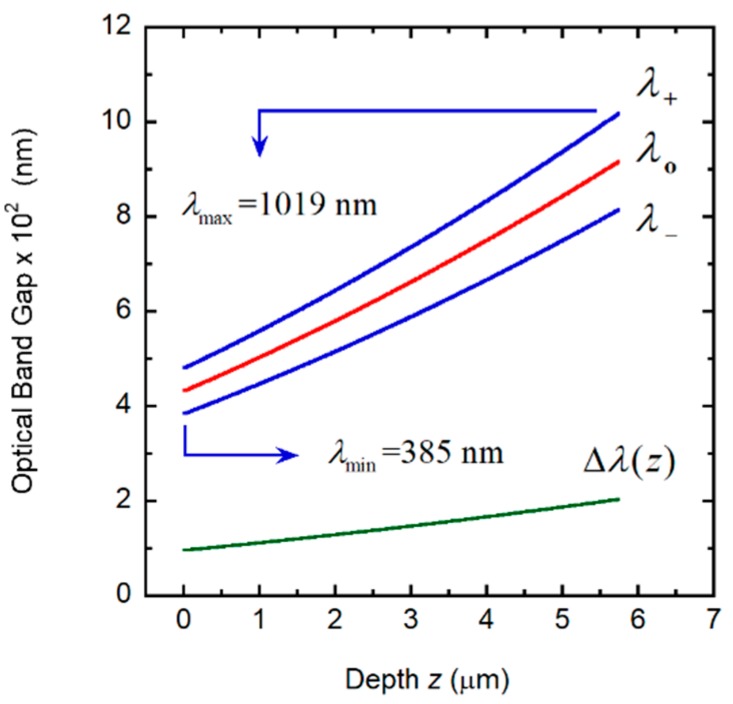
Depth dependence of the photonic band gap (Δ*λ*(*z*)), its spectral limits (*λ*_+_(*z*) and *λ*_−_(*z*)) and central position (*λ_o_*(*z*)). The spectral limits of the reflection band (*λ*_min_ and *λ*_max_) are also indicated.

In this section, the link between Berreman’s formalism and a PC characterization of the twisted structure is introduced by considering two chiral arrangements with assumed depth dependences of the pitches. A chiral twisted structure behaves like one-dimensional PC for that incident light whose circular polarization coincides with the chirality of the arrangement. At least for sufficiently thick structures, no photons with this chirality will be transmitted through the arrangement, for energies within the spectral location of the reflection band. As we have reported in previous works [31,33], when the pitch depends on the depth *z* through the arrangement, the spectral position of the PBG also depends on depth, i.e., both the spectral limits of the local band gap $\lambda_{\;-}=\lambda_{\;-}(z)$ and $\lambda_{\;+}=\lambda_{\;+}(z)$ depend on *z*. This fact explains why the reflection band is significantly broader than the PBG. For a linearly graded pitch, application of the formalism devised in [34,35], with the dispersion relation obtained from Hemholtz’s wave equation satisfied by the two orthogonal left- and right-handed circularly polarized components of the propagating electric field, gives for the spectral limits of the PBG:

(1) $$\lambda_{\;\pm }(z)=\frac{\sqrt{\bar{\varepsilon }}}{P_{m}}{\left[P(z)\right]}^{2}\frac{\sqrt{1-K^{2}}}{[1\mp \sqrt{1-(1-K^{2})(1+\Gamma )]}},$$

where $\bar{\varepsilon }=(\varepsilon_{e}+\varepsilon_{o})/2$, $\Gamma ={\left[P(z)\Delta P/\pi P_{m}H\right]}^{2}$ and $K=(\varepsilon_{e}-\varepsilon_{o})/(\varepsilon_{e}+\varepsilon_{o})$. The *z*-dependent spectral position of the PBG is $\lambda_{o}(z)=[\lambda_{+}(z)+\lambda_{-}(z)]/2$, and its local width is $\Delta \lambda (z)=\lambda_{+}(z)-\lambda_{-}(z)$. The effective width of the reflection band is obtained from the minimum value of $\lambda_{-}(z)$ and the maximum of $\lambda_{+}(z)$, i.e., $\min[\lambda_{-}(z)]\equiv \lambda_{\min}$ and $\max[\lambda_{+}(z)]\equiv \lambda_{\max}$. The center of the reflection band is at $\lambda_{o}=(\lambda_{\min}+\lambda_{\max})/2$, and its effective width is $\Delta \lambda =\lambda_{\max}-\lambda_{\min}$. For a helicoidal arrangement with a linearly graded pitch, the reflection band is characterized by the following limits:

(2) $$\lambda_{\min}=\sqrt{\bar{\varepsilon }}P_{m}\frac{\sqrt{1-K^{2}}}{[1+\sqrt{1-(1-K^{2})(1+\Gamma_{m})]}},\;\lambda_{\max}=\sqrt{\bar{\varepsilon }}\frac{P_{M}^{2}}{P_{m}}\frac{\sqrt{1-K^{2}}}{[1-\sqrt{1-(1-K^{2})(1+\Gamma_{M})]}},$$

where $\Gamma_{M}={\left(\Delta PP_{M}/\pi HP_{m}\right)}^{2}$ and $\Gamma_{m}={\left(\Delta P/\pi H\right)}^{2}$. The pitch values are of the order of tenths of a micrometer, and the thickness of the structures is around a few tens of micrometers. As a consequence of this, $\Gamma_{m}\ll 1$ and $\Gamma_{M}\ll 1$. The values of *λ*_min_ and *λ*_max_ can be approximated from the previous Equation (2) and the spectral position of the center of the reflection band, as well as its effective width becomes:

(3) $$\lambda_{o}=\frac{n_{e}P_{M}^{2}+n_{o}P_{m}^{2}}{2P_{m}},\;\Delta \lambda =\frac{n_{e}P_{M}^{2}-n_{o}P_{m}^{2}}{P_{m}},$$

with *λ*_min_ = *n_o_P_m_* and *λ*_max_ = *n_e_P_M_*^2^*/P_m_*. Equation (3) is the extended version of those equations valid for a single pitch structure (*λ_o_ = n_av_P_o_* and Δ*λ* = Δ*nP_o_*, respectively), when considering chiral chirped arrangements. It is conceptually erroneous to apply the equations *λ_o_ = n_av_P_o_* and Δ*λ* = Δ*nP_o_* when considering helical structures with graded pitches, as reported in [58], where *λ_o_* was estimated from *n_av_P_o_* to show that the depth-dependent pitch values obtained for the cuticle of silver-like *C. chrysargyrea* scarabs, based on the analysis of SEM images, are too small to justify the presence of a reflection band extending towards the NIR [35,58]. In these cases, the correct way to explain the effective width of the reflection band is by application of Equations (4)–(6) to obtain the minimum value of *λ*_−_ and the maximum value of *λ*_+_ for a specific depth dependence of the pitch. Namely,

(4) $$Q(z)\;=\;q(z)\;\left[1-\frac{z}{P(z)}\;\frac{dP}{dz}\right],$$

(5) $$\bar{\varepsilon }{\left(\frac{\omega_{\pm }}{c}\right)}^{2}\frac{1}{Q^{2}}\;=\;\frac{1\pm \sqrt{1-(1-K^{2})(1+{\bar{\Gamma }}^{2}/Q^{4})}}{1-K^{2}},$$

(6) $$\bar{\Gamma }\;=\;\frac{q(z)}{P(z)}\;\left[\frac{2z}{P(z)}\;{\left(\frac{dP}{dz}\right)}^{2}-2\;\left(\frac{dP}{dz}\right)-z\;\frac{d^{2}P}{dz^{2}}\right],$$

where *q*(*z*) = 2π*γ/P*(*z*), *c* is the speed of light in vacuum, and *ω* = 2π*c*/*λ* is the angular frequency of the incident radiation whose wavelength is *λ*, as described in [35]. When we apply Equation (3) to the linearly graded structure considered in Section 3.4, the following results are obtained: *λ_o_* = 702.5 nm and Δ*λ* = 636.8 nm, in good agreement with the reflection spectrum shown in Figure 7B. With no approximations, by application of Equation (1) or following the method reported in [35], the corresponding values are: *λ_o_* = 702.0 nm and Δ*λ* = 634.0 nm. Figure 8 shows the depth dependence of the local PBG Δ*λ*(*z*), its depth-dependent spectral limits *λ*_+_(*z*) and *λ_−_*(*z*) and its spectral position. 

**Figure 9:**
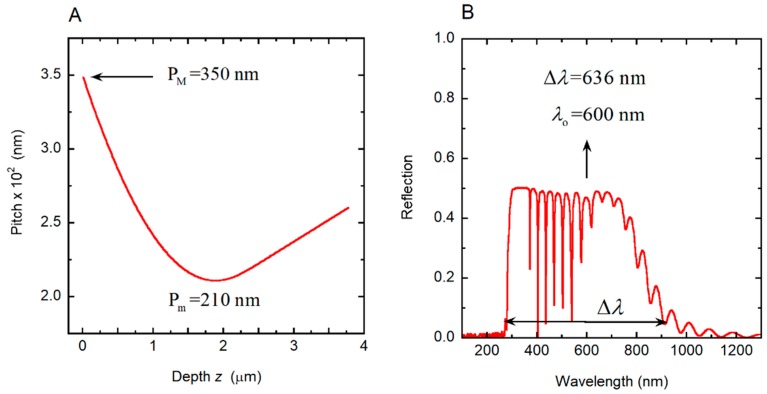
Dependence of pitch on depth *z* and corresponding reflection spectrum of a chiral structure. (**A**) Quasi-parabolic depth dependence of a helicoidal structure. Maximum (*P_M_*) and minimum (*P_m_*) values of the pitches are indicated. (**B**) Reflection spectrum of the structure by assuming normally incident non-polarized light. The reflection band is centered at *λ_o_* = 600 nm, and its width is Δ*λ* = 636 nm.

**Figure 10:**
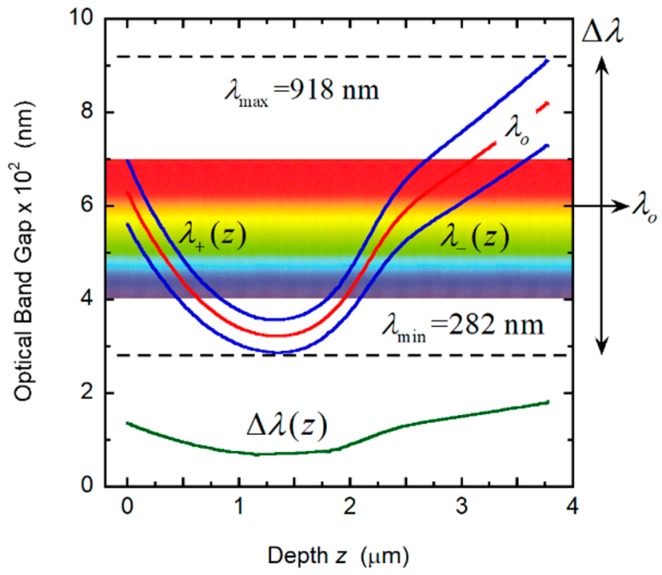
Variation with depth through the cuticle of the local photonic band gap (Δ*λ*(*z*)) and of its limits (*λ*_+_(*z*) and *λ*_−_(*z*)). The effective values of the central position of the band and its width are indicated as *λ_o_* and Δ*λ*.

The pitch in cuticles of some *Chrysina* scarab beetles has shown a predominantly quasi-parabolic depth dependence with a linear behavior in the deeper helicoidal layers (see Figure 6A in [35]). The minimum value of the pitch corresponds to the vertex of the parabola, with the maximum value close to the illuminated surface of the structure. With the aim of generating a pitch with these characteristics, the parabolic section is calculated from *P*(*z*) = *P_m_ + C* (*z − z_o_*)^2^, with *z_o_ = αH* (with *α* < 1) and *C* = Δ*P/*(*αH*)^2^ to ensure that *P*(*z* = 0) = *P_M_*. From *z*_1_ = *μH* (with *α* < *μ* < 1), a linear dependence holds: *P*(*z*) = *A + B*(*z − z_o_*) with *B* = 2*C*(*z*_1_ *− z_o_*) to match the slopes at *z*_1_ and *A = P_m_ + C*(*z*_1_ − *z_o_*)^2^ − *B*(*z*_1_ − *z_o_*) to ensure continuity of the *P*(*z*) function at *z = z*_1_.The following input values were used: *P_m_* = 210 nm, *P_M_* = 350 nm, *H* = 18*P_m_*, *n_o_* = 1.60, Δ*n* = 0.40. The values *α* = 0.5 and *μ* = 0.60 were assumed. Figure 9A shows the depth dependence of the pitch that we use to evaluate the spectral reflectance displayed in Figure 9B, which displays a broad structured reflection band from 282 to 918 nm. From Equation (1), with the application of Equations (4)–(6), the PC characterization depicted in Figure 10 is obtained. The visual silvery appearance of this hypothetical structure depends on Bragg reflection occurring in layers between a 0.5 and a 2.25 μm depth, as can be deduced from the analysis of the PC behavior shown in Figure 10. 

Color coordinates, evaluated with the standard procedure established by the Commission Internationale de l’Éclairage (CIE), which is based on the use of the color-matching functions as commented elsewhere [32,59] and by assuming the AM1.5 solar spectrum (which corresponds to a solar zenith angle of 48.2°), are *x* = 0.337 and *y* = 0.335 with a luminous reflection *Y* = 42.2. The corresponding RGB values are: R = 190, G = 169, and B = 164. The deeper layers contribute to the NIR reflection. 

The reflection band displays a ripple structure for wavelengths between 370 and 620 nm, which is produced by Bragg reflections at two ranges of depth: from the top of the structure to 0.8 μm of depth and from ≈1.9 to 2.6 μm of depth. For depths between ≈0.8 and 1.9 μm, the PBG is in the ultraviolet range, being responsible for the shoulder or narrow reflection band spectrally located between the reflection edge and the beginning of the ripple structure. The presence of this shoulder is determined by Bragg reflections at layers close to a 1.4 μm depth, where there is a range of depths with quasi-constant PBG. The presence of the ripple structure is determined by enhanced Bragg reflection due to a graded pitch with a parabolic or quasi-parabolic dependence on depth through some extended section of the elytron. Chiral chirped arrangements do not display any ripple structure in their reflection spectra.

### 3.6. Optical Characterization of the Cuticle of C. chrysargyrea Scarabs

**Figure 11:**
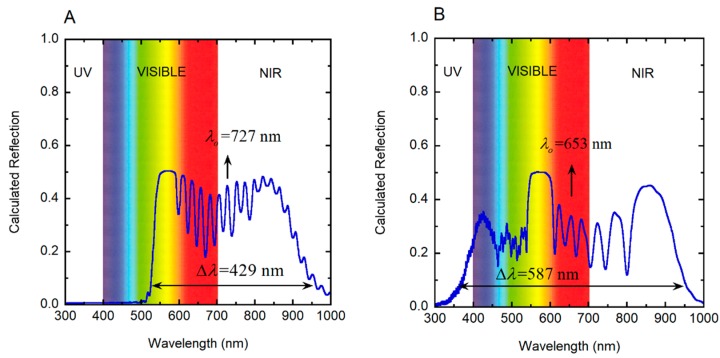
Calculated total reflection spectra of cuticles of (**A**) golden-like *C. chrysargyrea* and (**B**) silver-like *C. chrysargyrea* illuminated with non-polarized normally incident light. NIR: Near infrared; UV: Ultraviolet.

**Figure 12:**
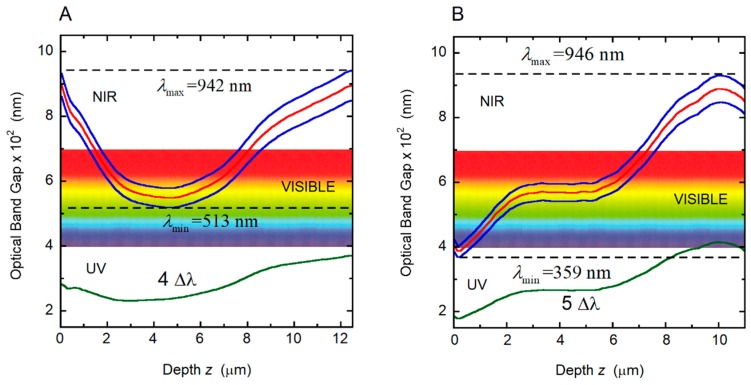
Variation with depth through the cuticle of the local photonic band gap (Δ*λ*(*z*)) and of its limits (*λ*_+_(*z*) and *λ*_−_(*z*), solid blue lines), for the two photonic crystals considered and correlated with the cuticles of (**A**) golden *C. chrysargyrea* and (**B**) silvery *C. chrysargyrea* scarabs whose calculated reflection spectra are displayed in Figure 11. The red line indicates the central position of the photonic band gap, *λ_o_*. NIR: Near infrared; UV: Ultraviolet.

This section is focused on modeling the reflection spectra by the elytra of both the golden- and silver-like *C. chrysargyrea* beetles. The correlation between optical properties and the cuticle’s morphology has been investigated during the last few years by several research groups, involving samples taken from beetles of the *Chrysina* genus. For example, the optical properties of seven species of scarab beetles (*C. macropus*, *C. peruviana*, *C. argenteola*, *C. resplendens*, *C. woodii*, *C. gloriosa*, and *C. chrysargyrea*) have been considered through ellipsometry measurements [21]. Our main purpose when modeling the reflection spectra will be to reproduce the main spectral features displayed in the optical measurements performed with normally incident non-polarized radiation. According to Equation (1), *λ*_min_ is proportional to *P_m_*. The minimum value of the pitch is correlated with the spectral position of the reflection edge. When using Berreman’s formalism to calculate reflection spectra, with *γ* = −1, the spectral positions of the reflection edge at short wavelengths and of the main reflection peaks are numerically sensitive to the angle of inclination (*β*). Moreover, this angle modifies the depth dependence of the pitch from apparent to real values through the relation *P*(*z*) → *P*(*z*)/cos*β* [35]. In this way, we optimized the *β*-value, obtaining 26° and 20° for the golden and the silvery *C. chrysargyrea*, respectively. With these *β*-values, the thicknesses of the twisted structures are 12.7 and 11.6 μm for the golden and silvery scarabs, respectively. The corresponding number of pitches is the same for both chiral structures, i.e., 29. The use by Finlayson et al. of a scaled depth-dependent pitch in their simulations of the co- and cross-polarized light reflections by the cuticle of a *C. resplendens* scarab can be alternatively interpreted as an implicit geometrical correction through a β-angle close to 25° [28]. Application of Berreman’s method required the optical constants of chitin and uric acid [60,61], incorporating their wavelength dependence. For the substrate of the chiral structure, we have assumed *n_s_* = *n_av_*. An optically homogenous layer of wax covers the structures whose refractive index *n_c_* is calculated from the model reported in [62]. The calculations were performed with *N* = 3 × 10^4^. The optimized volume fractions of uric acid are the same for both scarabs: *F_ua_* = 0.40, to obtain, as much as possible, similar relative heights of the main reflection peaks to those measured. The birefringence of chitin is small (Δ*n_c_* = 0.035 [63]) as compared with the birefringence of uric acid (Δ*n_ua_* = 0.31 [50]). The heights of the peaks in the calculated reflection spectra are determined by the effective birefringence. The presence of flattened shoulders or plateaus in the reflections’ spectra is an indication of high effective birefringence values. We use this fact to optimize the volume fraction of uric acid, to approach, as much as possible, the main spectral features displayed in the measured reflection spectra. The calculated total reflection spectra are shown in Figure 11 for the two *C. chrysargyrea* scarabs, which were considered in a previous study [35]. Only *β* and *F_ua_* are used as fitting parameters. Here, we report a better fitting of the reflection spectra, particularly that of the silvery *C. chrysargyrea* at short wavelengths, as well as the PC characterizations, which were not included previously. The primary reflection by the waxy coating of the cuticle, which is around 3% at short visible wavelengths, was not included in the displayed calculated reflection spectra. Comparing Figure 11A,B with the corresponding experimental measurements reported in Figure 2A,B, we observed some similarities in the spectra with respect to the main features: spectral position of the first reflection peak or shoulder, ripple structure or plateau around the middle of the reflection band, and decreasing values when approaching the NIR. Figure 11 shows the width of the reflection band (Δ*λ*) centered at *λ_o_*, whose values were calculated as explained in Section 3.5. Figure 12 shows the behaviors with depth through the cuticle of the local PBGs (Δ*λ*(*z*)), of its limit (*λ*_−_(*z*) and *λ*_+_(*z*)), and the limits of the effective width of the reflection bands (*λ*_min_ and *λ*_max_). From Figure 12A, we conclude that the visual golden appearance of the *C. chrysargyrea* is due to Bragg reflections at layers of the cuticle whose depths are between 1.7 and 8.0 μm: the yellow contribution is produced by reflections at depths between 2.5 and 7.0 μm, and the red component arises from reflections between 1.7 and 2.5 μm, and 7.0 and 8.0 μm. The layers located close to the surface, up to 1.7 μm, and those from 8.0 μm to the bottom of the structure are responsible for the NIR reflection. 

The reflection spectrum shows that the UV, violet, blue, bluish and green light are not reflected. This fact agrees with the information provided by the PC diagram in Figure 12A, where the minimum of the effective reflection band (λ_min_ = 513 nm) is above the mentioned spectral range. Regarding the silver-like *C. chrysargyrea*, there is a small fraction of UV light reflected by superficial layers, and all visible tones are reflected with the bluish colors emerging from layers beginning with the surface up to 1.5 μm (see Figure 12B). The green and yellow colors are due to Bragg reflections in layers between 1.5 and 6.5 μm in depth. The next micrometer in depth produces the red contribution, and the NIR reflected light emerges from layers located from 7.5 to 11.5 μm. Similar correlations, showing how to change the spectrum and corresponding color with the number of pitches contributing to the reflection, was performed for *C. aurigans* scarabs [33]. 

### 3.7. Optical Characterization of the Cuticle of *C. optima* Scarabs

**Figure 13:**
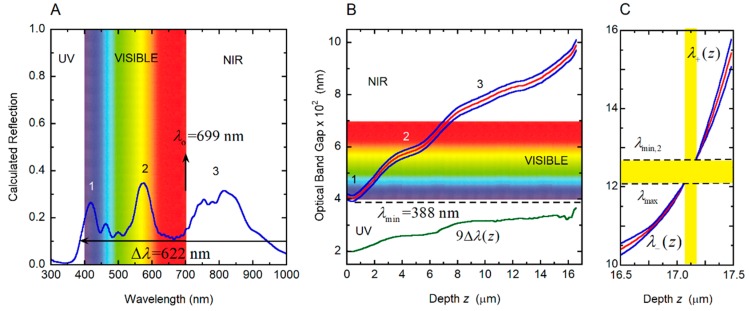
Reflection spectrum and photonic crystal characterization of the cuticle of the *C. optima* scarab. (**A**) Calculated total reflection spectra of the cuticles of *C. optima* illuminated with non-polarized normally incident light. (**B**) Variation with depth through the cuticle of the local photonic band gap (Δ*λ*(*z*)) and its limits (*λ*_+_(*z*) and *λ*_−_(*z*)), for the photonic crystal correlated with the cuticle of *C. optima* scarab. The limits of the reflection band, *λ*_min_ and *λ*_max_, are also indicated. Labels 1, 2, and 3 correlate the spectral positions of the reflection peaks with the local depth dependence of the pitch. (**C**) Photonic crystal characterization in the last micrometer of the twisted structure. NIR: Near infrared; UV: Ultraviolet.

The total reflection spectrum by the cuticle of a *C. optima* specimen has also been measured for normally incident non-polarized radiation (see Figure 2C). Three main peaks are distinguished, with some superimposed irregular spectral structure. A similar spectrum, in the wavelength range 450−1000 nm, has been previously reported [64]. The monotonous depth dependence of the pitch is not incorporated in the modeling of the reflection spectrum by these authors. Using the depth-dependent pitch shown in Figure 4C, the reflection spectrum of the chiral structure has been calculated, with *β* = 10° and with the average volume fraction of uric acid previously estimated as described in Section 3.3 (*F_ua_* = 0.17). The thickness of the structure is *H* = 17.5 μm, and the number of pitches is 40.5. Figure 13A shows the main three reflection peaks, with two smaller peaks between those spectrally located in the visible range. The spectral positions of the main peaks and those of the measured ones match very well. The fraction of uric acid estimated from the infrared absorbance spectra leads to peak heights close to the observed ones. The calculated spectrum was obtained by assuming a normal distribution in the depth dependence of the pitch around the mean value displayed in Figure 4C. The standard deviation was set to 5 nm, which is equal to the uncertainty in the determinations of *z*- and *P*(*z*)-values from SEM images. The relations between heights of the reflection peaks have been improved in the fitting by assuming a pitch dependence of the uric acid volume fraction, according to the assumed relation *f_ua_* = *C/P*(*z*) with the average volume fraction given by *F_ua_ = C/P_av_*. Then, *f_ua_ = F_ua_P_av_/P*(*z*) with the average value of the pitch evaluated from *P_av_* = (*P_m_ + P_M_*)/2cos*β* with *P_m_* = 250 nm and *P_M_* = 424 nm (see Figure 4C). The minimum value of *f_ua_* is 0.14, close to the back side of the structure, and the maximum value is 0.23, close to the illuminated surface. The mechanism involved in the incorporation of uric acid crystallites through the protein–chitin matrix of the chiral structure is basically unknown. The high birefringence required to approach the measured reflection spectra from modeling indicates that the orientation of the crystallites is not at random. The protein–chitin matrix is probably permeated by liquid uric acid, perhaps containing nanocrystals, being conducted throughout the submicron-sized pore canals distributed across the chiral structure and nearly perpendicular to the planes containing the oriented chitin nanofibrils. This uric acid could be conducted from each pore canal to the interstitial space between chitin nano-fibril, where it could grow to form oriented larger crystals. The larger the pitch of the structure is, the larger the available space between planes of chitin nanofibrils. This means that as the pitch increases with depth, it would be expected to have larger embedded uric acid crystallites. Beyond a certain size, around 10 nm, the larger the size of the crystallites, the larger the amount of space free of uric acid, i.e., the space between crystallites. Consequently, the volume fraction of uric acid will decrease as the pitch of the structure increases. It is based on this analysis that we assumed an inverse relation between the volume fraction of uric acid and the spatial period or pitch.

According to the PC diagram in Figure 13B, the reflection edge is displayed at *λ*_min_ = 388 nm. The PBG is displayed up to *λ*_max_ = 1209 nm, as shown in Figure 13C, which depicts the PC characterization in the last micrometer of the twisted structure. The effective width of the reflection band is then Δ*λ* = 821 nm. In this case, a novel feature is observed: the PBG disappears for thicknesses between 17.06 and 17.17 μm, and for wavelengths between *λ*_max_ = 1209 and *λ*_min,2_ = 1270 nm. This *λ*_min,2_ becomes the spectral edge of a secondary PBG. The first reflection peak, labeled 1 within the violet-blue colors (Figure 13B), is due to the localized quasi-parabolic dependence of the pitch through the superficial layers whose depths are from the illuminated surface up to 1.8 μm in depth. The second peak, labeled 2 and with a maximum in the yellowish colors, is associated with the shoulder displayed in the depth dependence of the pitch for depths between 3.0 and 5.5 μm. The broader structured peak in the NIR, labeled 3, is due to the shoulder of the pitch’s depth dependence for *z*-values between 8 and 13 μm.

## 4. Conclusions

The optical properties of cuticles of *Chrysina* scarab beetles have been considered from spectrophotometric measurements and modeled with Berreman’s formalism, which incorporates the anisotropy in the optical constants of the materials: the natural polymer chitin nanofibrils and the uric acid high birefringent embedded crystals. In addition to the optical constants with their wavelength dependences, variations with depth of the pitches characterizing the chiral structures found through the cuticles have been provided to the model. This dependence has been obtained from analysis of SEM images of cross-sections of the cuticles. Fourier-transform infrared and Raman spectroscopies were used to characterize the composition of the cuticles, particularly to corroborate, with a high degree of certainty, the presence of uric acid in the cuticle of *C. optima*. With only one or two fitting parameters (uric acid volume fraction and inclination angle), the main spectral features of the measured total reflection spectra are reproduced from the application of the mentioned radiative transfer matrix formalism, when considering reflection spectra by cuticles of golden *C. chrysargyrea*, silvery *C. chrysargyrea*, and silvery *C. optima* scarab beetles. A method described previously in [35] has been devised to characterize the PC behavior of the twisted structures, for that circular polarization component matching the chirality of the arrangement. Using this method, the PBG is obtained as a function of the depth through the cuticle, its spectral limits, as well as the limits of the broad reflection band displayed both in the measured reflection spectra and in the calculated ones. We expect that the developed methodology reported in this article can be used to predict the optical properties of other natural systems with chirality and simulated or synthetized functional chiral arrangements.
